# Cross-talk between CD8+ T cells and natural killers: the role of mitochondrial Aa2+ transport

**DOI:** 10.1186/2051-1426-2-S3-P189

**Published:** 2014-11-06

**Authors:** Roman Uzhachenko, Shawn Goodwin, Menaka Thounaojam, William Hofmeister, Anil Shanker

**Affiliations:** 1 Meharry Medical College, Nashville, TN, USA; 2 University of TN Space Institute, Nashville, TN, USA; 3 Meharry Medical College School of Medicine / Vanderbilt-Ingram Cancer Center, Nashville, TN, USA

## 

Unraveling complex interactions between immune cells is a key to the development of new strategies for immunotherapy. In the present study, we investigated functional outcome of bidirectional interaction between activated CD8+T and naive natural killer (NK) cells. We found that phorbol 12-myristate 13-acetate (PMA)/Ionomycin (Io)-stimulated CD8+T cells form multiple intercellular contacts with naive NK lymphocytes. Co-culture of activated T cells with naïve NK cells results in the selective down-regulation of CD25 molecule in T cells while elevating CD25 and CD69 expression on naive NK cells. Further, CD8+T and NK cells cross-regulate mitochondrial homeostasis including calcium transport. This effect is dependent on both cytokines and intercellular contacts, and partially involves natural killer group 2 member D (NKG2D) receptor activation. Data also suggest that activated CD8+T cells might directly transfer mitochondria and activation molecules such as CD25 and CD69 to naive NK cells. Alterations in phosporylation status of multiple signaling proteins during CD8+T/NK interaction suggest a functional remodeling whereby NK cells shift activated CD8+T cells towards T central-memory (TCM) phenotype and activated CD8+T lymphocytes alter naive state of NK cells towards effector/regulatory phenotype. Inhibition of mitochondrial Ca2+ uptake (mCU) or Na+/Ca2+ exchanger (mNCE) with Ru360 and CGP37157 respectively mimicked observed alterations in CD8+ and NK cells upon their interaction. These data suggest a potential role of mitochondrial Ca2+ homeostasis in the acquisition of mixed activation/regulatory phenotype by NK cells during T cell-NK cell interaction. We believe that understanding the mechanisms of CD8+-NK interplay will help to develop new approaches for cellular immunotherapy.

